# Failure of the Condyle-C1 Interval Method to Diagnose Atlanto-occipital Dislocation in the Presence of an Associated Atlanto-axial Dislocation: A Case Report

**DOI:** 10.7759/cureus.2486

**Published:** 2018-04-16

**Authors:** Mohamed Abouelleil, Daanish Siddique, Nader S Dahdaleh

**Affiliations:** 1 University of Illinois College of Medicine, Champaign, USA; 2 Neurological Surgery, Northwestern University Feinberg School of Medicine

**Keywords:** atlanto-occipital dislocation, atlanto-axial dislocation, occipital condyle-c1 interval, craniocervical injury

## Abstract

Atlanto-occipital dislocation (AOD) is a craniocervical injury that has serious neurological consequences and is often fatal. High-speed blunt trauma, such as motor vehicle accidents, that extend and put traction on the head can cause this injury. The current recommendation for diagnosis is to measure the condyle-C1 interval (CCI) using a computed tomography (CT) scan in the coronal plane and more recently in the sagittal plane. We report the case of a patient who suffered a motor vehicle accident and had concomitant AOD and atlanto-axial dislocation. In this particular case, the CCI method failed to diagnose AOD and the diagnosis was made using the basion-dens interval (BDI) and other methodologies, as well as the presence of ligamentous disruption at the craniovertebral junction (CVJ) on magnetic resonance imaging (MRI).

A 19-year-old female suffered a motor vehicle accident in which she was ejected from the car. Her neck was immobilized on the scene and she was brought to the emergency department complaining of neck pain. CT of the cervical spine showed concomitant atlanto-occipital and atlanto-axial dissociation. MRI of the cervical spine confirmed the diagnosis with total ligamentous disruption at the CVJ and distraction of the atlanto-axial joints bilaterally. While the CCI was normal, the BDI was diagnostic of AOD.

The current recommendations for using the CCI interval method may not diagnose AOD in the presence of associated atlanto-axial dislocation. Other methodologies should be employed including BDI and basion-axial interval (BAI) as well as MR imaging showing ligamentous disruption.

## Introduction

Atlanto-occipital dislocation (AOD) is a craniocervical injury that can lead to serious neurological consequences and fatalities. Commonly, patients suffer respiratory arrest and quadriplegia due to injury to the cervico-medullary junction. The mechanism of injury is extension and traction of the head, which typically occurs in high-speed blunt trauma such as motor vehicle accidents. A study conducted at a level 1 trauma center reviewed 2,616 traumatic cervical spine computed tomography (CT) scans over a five-year period and found the incidence of this mechanism of injury to be 0.2% reflecting that these injuries are not common [1]. Improvements in pre-hospital cervical spine stabilization and management combined with early recognition and diagnosis have led to improvements in outcomes and survival.

The current recommendation for diagnosis is to measure the condyle-C1 interval (CCI) using a CT scan in the coronal plane and more recently in the sagittal plane [2-4]. This method has been proven to provide the diagnosis with very high sensitivities and positive predictive values approaching 100%. A value of 2.5 mm is diagnostic of this injury. There are other methods that have been used to diagnose AOD including basion-dens interval (BDI), basion-axial interval (BAI), X-lines of Lee, Powers ratio, with variable sensitivities [2]. We report a case of a patient who suffered a motor vehicle accident and had concomitant AOD and atlanto-axial dislocation. In this particular case, the CCI method failed to diagnose AOD and the diagnosis was made using other methodologies including the BDI and the presence of ligamentous disruption at the CVJ on magnetic resonance imaging (MRI) scanning.

## Case presentation

**Figure 1:**
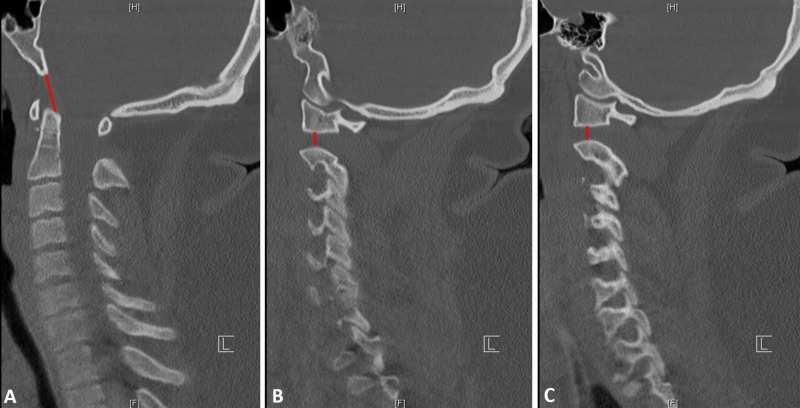
Atlanto-occipital dissociation Sagittal (A) and bilateral parasagittal (B and C) computed tomography (CT) scans showing atlanto-occiptial dissociation diagnosed by the basion interval index > 12 mm (red line A) and atlanto-axial dislocation with increased distraction of the atlanto-axial joints bilaterally (red lines B and C). Note that the condyle-C1 (CCI) interval was normal (B and C).

**Figure 2:**
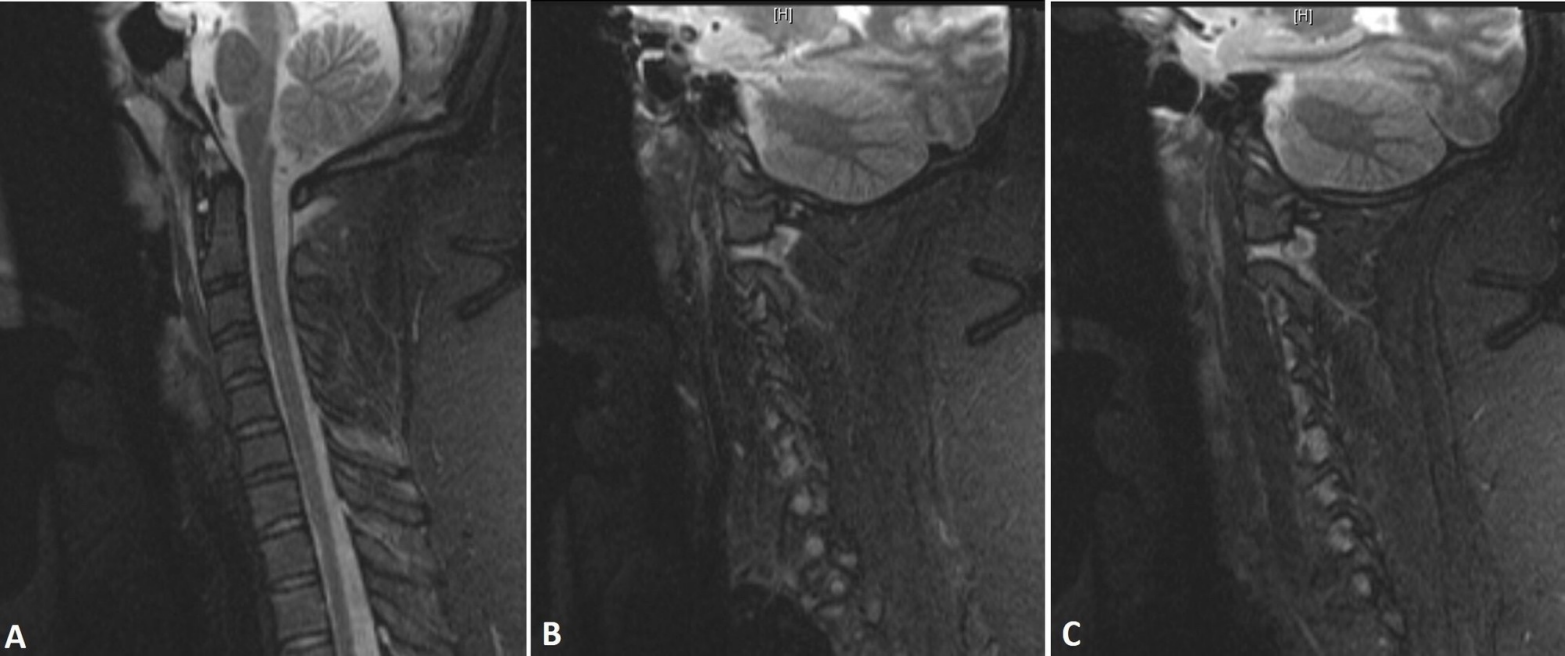
Ligamentous disruption Sagittal (A) and bilateral parasagittal (B and C) STIR MRI sequences showing disruption of the apical ligament and abnormal BDI (A) as well as increased signal intensity at the level of the atlanto-axial joints with distraction and an increase in signal intensity at the level of the condyle C1 joints bilaterally (B and C). STIR: short tau inversion recovery; MRI: magnetic resonance imaging; BDI: basion-dens interval.

**Figure 3:**
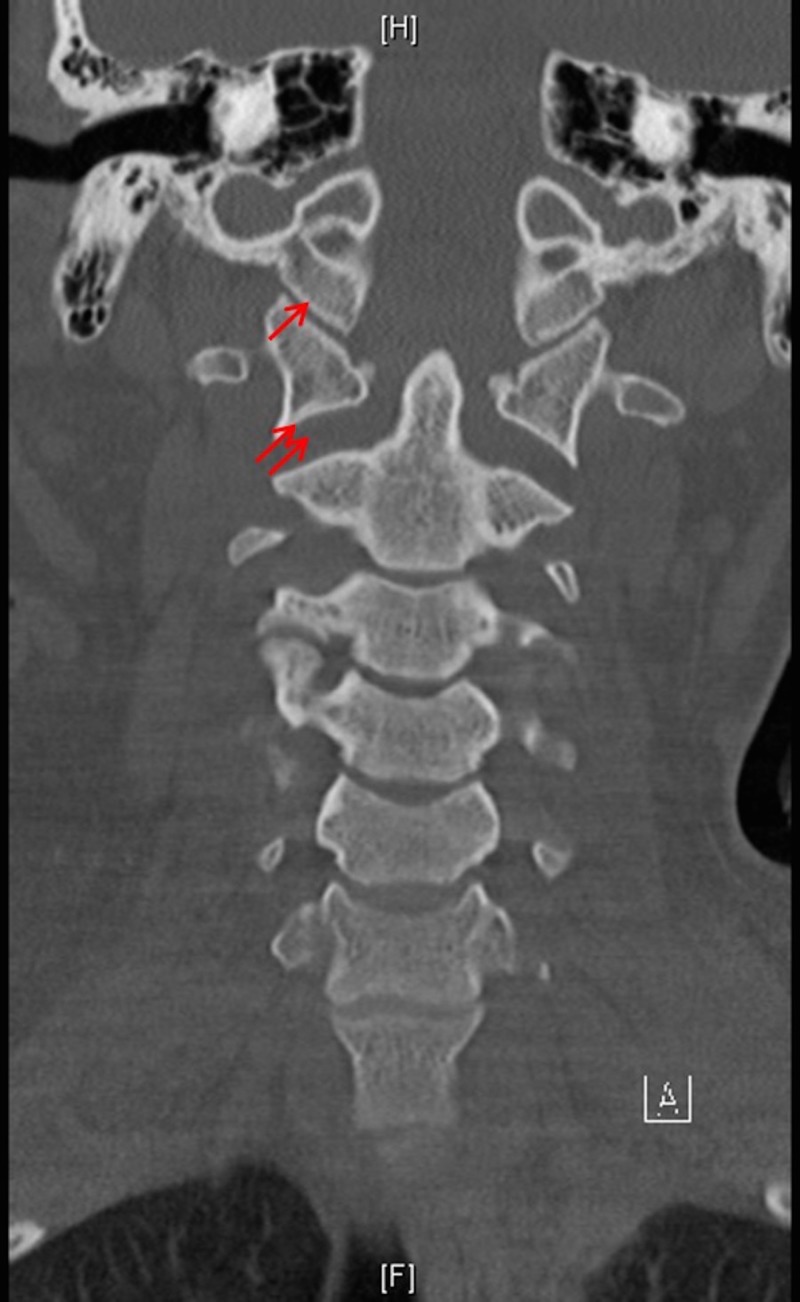
Atlanto-axial joint Coronal reconstructed computed tomography (CT) scan showing normal condyle-C1 interval (CCI) (arrow) and distracted atlanto-axial joints (double arrows).

A 19-year-old female suffered a motor vehicle accident causing her to be ejected from the car. Complete spinal precautions were followed at the scene and her neck was immobilized with a rigid collar. The patient then was transported to our emergency room. She was complaining of neck pain. Her vital signs and neurological examination were normal. She was found to have an associated left comminuted femur fracture. CT of the cervical spine showed concomitant atlanto-occipital and atlanto-axial dissociation (Figure 1). MRI of the cervical spine confirmed the diagnosis with total ligamentous disruption at the craniovertebral junction (CVJ) and distraction of the atlanto-axial joints bilaterally (Figures 2-3). While the CCI was normal (1.2 mm), the BDI was 19 mm, which is diagnostic of AOD.

**Figure 4:**
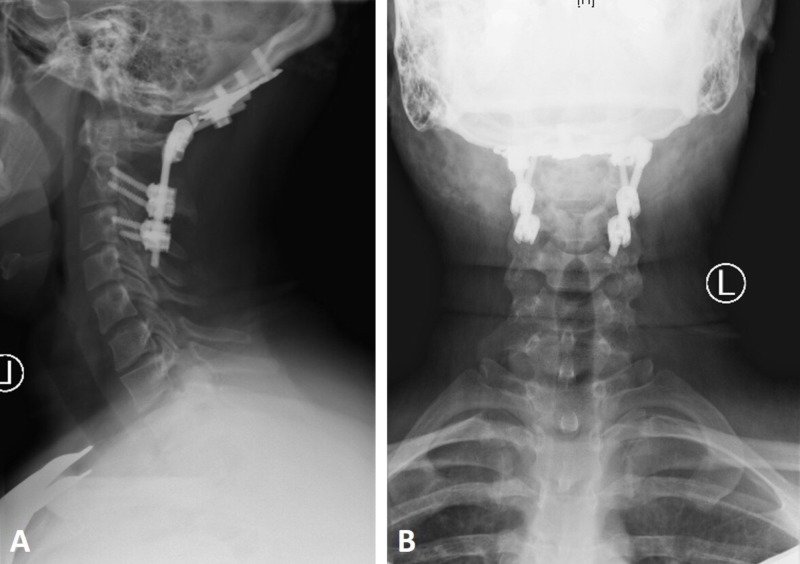
Occipitocervical fusion Lateral and anteroposterior cervical x-rays showing occipitocervical fusion from the occiput to C3 showing appropriate alignment at the craniovertebral junction (CVJ).

The patient was immobilized with a crown-halo vest and a posterior occipitocervical fusion was performed urgently (Figure 4). The patient then was discharged on a rigid collar and followed up in the clinic. During her one year appointment, the patient was neurologically normal, reported no neck pain, and denied any dysphagia.

## Discussion

The CVJ is made of the occiput, atlas, and axis. While the orientation of the atlanto-occipital and atlanto-axial joints determine the direction of motion accounting for at least 50% of motion in all planes, the complex ligamentous attachments determine biomechanical stability [5].

AOD is a severe injury that is associated with high mortality rates. There are several ways of diagnosing AOD, including the BAI [5], BDI [6], Powers ratio [7], Sun ratio [8], Wackenheim line [9], Lee X-lines [10], and the occipital CCI [11-12]. Diagnosing AOD is best done with CT scan in the coronal view [2]. A revised CCI was done by Gire, Roberto, Bobinski, and Klineberg [3], and stated that AOD can be accurately diagnosed by a unilateral dislocation or dissociation in the sagittal plane of the C1 joint of 2.5 mm. The revised CCI had a 100% sensitivity, specificity, positive predictive value, and negative predictive value and has been confirmed in other studies [2,4].

In a study by Campo, Kalb, and Baron [4], the CCI was proposed to be 1.5 mm as the upper limit of the normal values with a 100% sensitivity and specificity. Other studies report 2.5 mm as the cut off [2-3]. The condylar sum values were also suggested to change from 5.0 mm to 3.0 mm in order to raise suspicion for AOD. Due to natural changes that occur with aging, instability of the CVJ can occur with minimum widening as seen in a patient with a CCI of 1.6 mm that was later confirmed during surgery.

While the CCI was found to be the most reliable for AOD, we have found that to be invalid in our patient. The patient had a normal CCI and hence this method failed to diagnose AOD. The BDI method was positive, hence making the diagnosis. The reason was that the patient had an associated atlanto-axial dissociation or dislocation. While AOD was clear with disruption of the alar and apical ligaments anteriorly, the atlanto-occipital joint was well aligned and the dislocation was at the level of the atlanto-axial joints with disruption of that joint leading the widening and distraction (Figure 2). The patient was hence managed with occipitocervical fusion and had an excellent outcome.

## Conclusions

The CCI is the most sensitive and specific method to diagnose AOD. However, this method may not be sufficient in the presence of a concomitant atlanto-axial dislocation such as in our patient. While our patient was treated with an occipitocervical fusion with a great outcome, we implore the usage of other methodologies to diagnose AOD such as BDI, BAI, as well as MR imaging showing ligamentous disruption.
